# WTAP Maintains Alternative Activation of Macrophages by Promoting IDH1-Mediated α-ketoglutarate Production

**DOI:** 10.7150/ijbs.115672

**Published:** 2025-08-22

**Authors:** Qianqian Xu, Jing Zhang, Yuan Zou, Longmin Chen, Fei Sun, Xi Luo, Ting Wang, Yang Li, Shu Zhang, Fei Xiong, Qilin Yu, Ping Yang, Quan Gong, Shi-Wei Liu, Cong-Yi Wang

**Affiliations:** 1 Department of Respiratory and Critical Care Medicine, the Center for Biomedical Research, NHC Key Laboratory for Respiratory Diseases, Tongji Hospital, Tongji Medical College, Huazhong University of Science and Technology, Wuhan, China.; 2 Department of Rehabilitation, Tongji Hospital, Tongji Medical College, Huazhong University of Science and Technology, Wuhan, China.; 3 Department of Immunology, School of Medicine, Yangtze University, Jingzhou, China.; 4 Shanxi Bethune Hospital, Shanxi Academy of Medical Science, Tongji Shanxi Hospital, Third Hospital of Shanxi Medical University, the Key Laboratory of Endocrine and Metabolic Diseases of Shanxi Province, Taiyuan, China.; 5 The Center for Biomedical Research, Tongji Hospital Research Building, Tongji Hospital, Tongji Medical College, Huazhong University of Science and Technology, Wuhan, China.; 6 Diabetes Research Center, Qatar Biomedical Research Institute, Hamad Bin Khalifa University, Doha, Qatar.

**Keywords:** obesity, macrophage, WTAP, m^6^A modification, IDH1, α-ketoglutarate

## Abstract

**Background**: *N*^6^-methyladenosine (m^6^A) modification plays a crucial role in various physiological processes by regulating mRNA biology. However, the exact impact of m^6^A modification on macrophages in adipose tissues under obese settings remains to be further elucidated.

**Methods**: We established macrophage-specific *Wtap*-deficient mice to explore the effects of *Wtap* on obesity and metabolic disorders induced by high-fat diet (HFD) in mice. The molecular targets were explored by MeRIP-qPCR, and the metabolomic assays were performed to detect the alteration of relevant metabolites.

**Results**: Wilms tumor 1-associated protein (WTAP), one of the m^6^A “writers”, was downregulated in adipose tissue macrophages (ATMs) from obese individuals and negatively correlated with clinical metabolic traits. Depletion of *Wtap* in mouse macrophages exacerbated the metabolic consequences of high-fat diet (HFD) induced obesity. Additionally, energy expenditure and adipose beiging were considerably lower in *Wtap*-deficient mice in response to cold exposure. Mechanistic study revealed that WTAP-mediated m^6^A modification of isocitrate dehydrogenase 1 (*Idh1*) transcripts enhanced its stability and translation in macrophages leading to α-ketoglutarate (α-KG) production. Alpha-KG further supported alternative activation of macrophages by metabolic reprogramming.

**Conclusions**: Our data support that *Wtap* modulates HFD-induced macrophages through interfering with the IDH1-α-KG axis, and highlight the importance of WTAP-mediated m^6^A modification in maintaining alternative macrophage activation, proposing potential targets for the regulation of obesity and related metabolic diseases.

## Introduction

Chronic inflammation in adipose tissue is a crucial factor predisposing to the development of obesity and its related metabolic disorders 1, which is featured by the infiltration and activation of immune cells coupled with secretion of pro-inflammatory mediators 2. Particularly, macrophages are the most abundant immune cells in adipose tissues 3. In response to metabolic cues, adipose tissue macrophages (ATMs) present in a spectrum of heterogeneous activation states, exerting significant modulatory effects on metabolic outcomes 4. During obesity progression, macrophages accumulate in crown-like structures (CLSs) surrounding the dying adipocytes, adopt an inflammatory phenotype and mediate the development of insulin resistance. In contrast, lean fat ATMs are uniformly dispersed, typically express alternatively activated markers and release anti-inflammatory cytokines, by which they maintain the homeostasis of adipose tissue by resolving inflammation 5. In addition to the anti-inflammatory properties, previous studies including ours also support that alternatively activated macrophages may enhance adipose browning and beiging process, thereby regulating adaptive thermogenesis and energy expenditure 6, 7. In this respect, shifting macrophages toward alternatively activated phenotype may help to establish a healthy metabolic state.

*N*^6^-methyladenosine (m^6^A) is the most common and abundant messenger RNA (mRNA) modification and defined as one layer of the epitranscriptomes 8. In mammals, the m^6^A modification is deposited by the methyltransferase complex known as “writers”, composed of methyltransferase-like 3 (METTL3), methyltransferase-like 14 (METTL14), and Wilms tumor-1-associating protein (WTAP), among which METTL3 is responsible for catalyzing m^6^A formation, METTL14 is in charge of binding target mRNA, and WTAP is involved in the localization of the complex into nuclear speckles 9. By contrast, the methylation can be removed by two demethylases (erasers): fat mass and obesity-associated gene (FTO) and AlkB homolog 5 (ALKBH5). These modifications are recognized and interpreted by “readers”, including the YT521-B homology domain family (YTHDF) proteins and the insulin-like growth factor 2 mRNA binding proteins (IGF2BPs) 10. m^6^A participates in the regulation of various cellular phenotypes and biological processes, with a broad influence in mRNA splicing, stability, nuclear export, and translation 11. Therefore, a growing number of studies revealed that alterations in m^6^A contribute to the dysfunction of adipose tissue, liver, islets, and cardiovascular system, thereby exacerbating the incidence and development of metabolic disorders 12-15. However, unlike other m^6^A effectors, the role of WTAP in pathological conditions is relatively less appreciated, particularly in the setting of metabolic disorders.

Herein in this report, we noted that ATMs originated from obese subjects and high-fat diet (HFD) challenged mice are featured by the repressed WTAP expression. Macrophages deficient in *Wtap* significantly exacerbated adipose inflammatory responses along with metabolic dysfunction following the insult of metabolic stress. Mechanistically, loss of *Wtap* in macrophages dampened isocitrate dehydrogenase 1 (IDH1) mRNA stability and protein expression by decreasing m^6^A modification. The reduced IDH1 expression then led to the decreased production of α-ketoglutarate (α-KG), a critical metabolite that governs the macrophage alternative phenotype and energy balance 16, 17. Collectively, our findings shed new light on the epitranscriptomic regulation of macrophage program in obese settings *via* WTAP-mediated m^6^A modification.

## Materials and Methods

### Mouse models

The *Wtap*^flox/flox^ (*Wtap*^f/f^) mice in the C57BL/6 background were generated by targeting exon 3 using the CRISPR/Cas9 technique as described (Fig. 2A). The *LysM*-Cre transgenic mice in the C57BL/6 background were purchased from the Jackson's Laboratory (Bar Harbor, ME, USA). *LysM*-Cre *Wtap*^f/f^ mice were generated by crossing the *LysM*-Cre mice with *Wtap*^f/f^ mice for specific deletion of *Wtap* in macrophages. All mice were bred under specific pathogen-free (SPF) conditions with a 12-hour light/dark cycle, at a temperature of 20-24 °C and humidity of 45-65% in the Experimental Animal Center of Tongji Hospital. In the HFD model, 8-week-old *LysM*-Cre *Wtap*^f/f^ mice and their littermates were continuously provided with a high-fat diet (60% kcal fat; D112492, Research Diet, Gardners, USA) for 12 weeks. All tissues and blood samples were collected at the same time. In the cold stimulation model, one mouse per cage was exposed to a consistent temperature of 4 °C at 8 weeks of age, and the initial changes of the body temperatures were recorded. All experimental procedures were approved by the Animal Care and Use Committee of Tongji Hospital (TJH-202206021) in accordance with the guidelines of the National Institutes of Health (NIH).

### Human samples

The adipose tissues used in this study were derived from non-obese and obese individuals. Detailed information regarding clinically relevant indicators was provided in Table S1. All subjects exhibited no apparent chronic infection, or history of myocardial infarction. Informed consent was obtained from all the study participants. The human studies were conducted following the NIH guidelines and received approval from the Institutional Review Board (IRB) of Tongji Hospital (TJ-IRB20160601 and TJ-IRB20160602), as well as the Ethics Committee (2022-S-62) of Chengdu Third Hospital.

### Western blot and RT-qPCR analysis

Adipose tissues and cultured cells were lysed on ice using the RIPA buffer (Beyotime, Shanghai, China) containing protease inhibitor cocktail (Roche, Indianapolis, IN, USA). The homogenized proteins were separated by 10% SDS-PAGE gels and transferred onto the PVDF membranes. The membranes were then incubated with specific antibodies at a 1:1000 dilution to analyze the protein levels. The primary antibodies used in the research were purchased from Proteintech (Wuhan, China), which included WTAP (10200-1-AP), Arg1 (16001-1-AP), IDH1 (12332-1-AP), IGF2BP1 (22803-1-AP), IGF2BP2 (11601-1-AP), IGF2BP3 (14642-1-AP), Ucp1 (23673-1-AP), Lamin B (12987-1-AP) and β-Actin (66009-1-Ig). The relative gray values of the western blot bands were quantified using ImageJ software (version 1.46r).

Quantitative real-time PCR was performed using the SYBR Premix Ex Taq (TaKaRa, Tokyo, Japan) as previously described 18. The relative expression levels were calculated for each target gene using the *2^-ΔΔCt^* method. All primers in this study were listed in Table S2.

### Flow cytometry analysis

The cells were washed with PBS and incubated with indicated antibodies for 30 min on ice for surface staining. For intracellular markers, the cells were fixed at 4 °C for 30 min using Transcription Factor Buffer Set (562574; BD Biosciences, San Diego, CA, USA). The cells were than stained in Permeabilization Wash Buffer (421002; Biolegend, San Diego, CA, USA) with the relevant antibodies at 4 °C for 30 min. The following antibodies were used for the studies: PE anti-mouse F4/80 (123110), FITC anti-mouse CD11c (117306), APC anti-mouse CD206 (141708), PE/Cyanine7 anti-mouse CD86 Antibody (105014), FITC anti-mouse CD4 (100406), PerCP anti-mouse CD8a (100732), AlexaFluor 647 anti-mouse/rat/human Foxp3 (320014), PE anti-mouse/human CD44 (103008), and APC anti-mouse CD62L (104412), and Pacific Blue anti-mouse I-A/I-E (107620) from Biolegend (San Diego, CA, USA), and PE-Cy7 anti-mouse CD11b (552850) from BD Biosciences (San Diego, CA, USA). The antibody was at 1:200 dilution. The Flow cytometry data were obtained from MACSQuant^TM^ (Miltenyi Biotec, Auburn, CA, USA) and analyzed by the FlowJo software (v10.5.3).

### Histological and immunostaining analysis

The tissues were fixed in 4% paraformaldehyde for 24 h at room temperature, and then embedded in paraffin and cut into sections. Deparaffinization and Hematoxylin and Eosin staining were performed following previously established methods 19. For immunostaining, the sections were dipped in antigen retrieval buffer (10 mM sodium citrate, 0.05% Tween-20, pH 6.0) after deparaffinization, and subjected to high-pressure treatment for 7 min. The sections were then washed with PBS. The tissue sections were subsequently blocked with 5% donkey serum for 1 h at room temperature, followed by incubating with F4/80 (sc-377009; Santa Cruz Biotechnology, Santa Cruz, CA, USA) antibody for 16 h at 4 °C. On the following day, the sections were stained with an AlexaFluor 488-conjugated anti-mouse IgG antibody (Jackson ImmunoResearch Laboratories, West Grove, PA). Images were captured under a fluorescence microscope (OLYMPUS Upright microscope BX53, Olympus Corporation). For Oil Red O staining, the frozen liver sections were handled according to the established techniques 20.

### Seahorse assay

After the stimulation of mature BMDMs, metabolic activity was measured for OCR by a Seahorse XFe24 analyzer (Agilent Technologies, Santa Clara, CA, USA). Before conducting the experiment, the cell handling method remained consistent with previous practices. The OCR was measured after treatment with 1.5 μM oligomycin, 1.0 μM FCCP, and 0.5 μM antimycin A/rotenone. Subsequently, the data were normalized by protein concentration, and in-depth analysis was conducted using the XFe Wave software (Agilent Technologies, Santa Clara, CA, USA) as instructed.

### MeRIP-qPCR and RIP-qPCR

Two hundred μL of total RNA was sheared into fragments by incubating at 94 °C for 4 min, and then purified on ice after termination with EDTA. Next, the fragmented RNAs were incubated with an m^6^A antibody- or mouse IgG-conjugated Protein A/G Magnetic Beads at 4 °C for 16-19 h. Finally, the bound methylated RNA on the magnetic beads was competitively eluted out using a high concentration of ethanol at low temperature. All reagents and consumables should be guaranteed to be free of any RNA enzymes. The precipitated RNA was then subjected to qPCR analysis using specific primers. The m^6^A-IP results were normalized by the corresponding inputs in each sample based on the Cq values. The primer sequences for the m^6^A site of *Idh1* were as follows: forward 5'-GCT TGA TAA CAA TAC TGA GCT CAG C-3'; and reverse 5'-TTA AAG TTT GGC CTG AGC TAA TTT G-3'

### RNA stability assay

Actinomycin D (HY-17559; MedChemExpress, Shanghai, China) was added to the cells at a final concentration of 5 μM, and the cells were harvested before or at the indicated time points after adding Actinomycin D. Total cellular RNA was extracted using standard protocols and quantified by RT-qPCR analysis as described earlier.

### m^6^A dot blot

The RNA samples (200 μg for each sample) were heated at 95 °C for 3 min to eliminate secondary structures, followed by rapid cooling on ice. Next, 2 μL of each RNA sample was applied to the surface of a nitrocellulose (NC) membrane and allowed to dry. The RNA was then cross-linked to the membrane by exposure to UV light for 30 min. Subsequently, the membrane was incubated overnight at 4 °C with an m^6^A antibody. The next day, the membrane was washed three times with TBST, each wash lasted 10 min, and then incubated with the appropriate secondary antibody at room temperature for 1 h. The signal spots were detected using an enhanced chemiluminescence (ECL) kit after washing off the secondary antibody. Finally, methylene blue staining was performed as a loading control.

### Luciferase reporter assay

HEK293T cells were evenly seeded into a 24-well plate, followed by transfection with the wild-type or mutant *Idh1* reporter plasmids using the Lipofectamine 3000 (L3000015; Invitrogen, Carlsbad, CA) according to the instructions. The cells were collected after 48 h of transfection, and the relative luciferase activities were determined with a Dual Luciferase Reporter Gene Assay Kit (11402ES60, Yeasen, Shanghai, China) according to the manufacturer's instructions.

### Statistical analysis

All experimental data in this report were presented as mean ± SEM and were processed by the GraphPad Prism 5 software (GraphPad Software Inc., San Diego, CA). Differences between groups were assessed using unpaired two-tailed Student's *t* tests or one-way ANOVA. A *p* value less than 0.05 was considered statistical significance.

## Results

### Reduced WTAP expression in ATMs correlates with poor metabolic parameters

**Figure 1:**
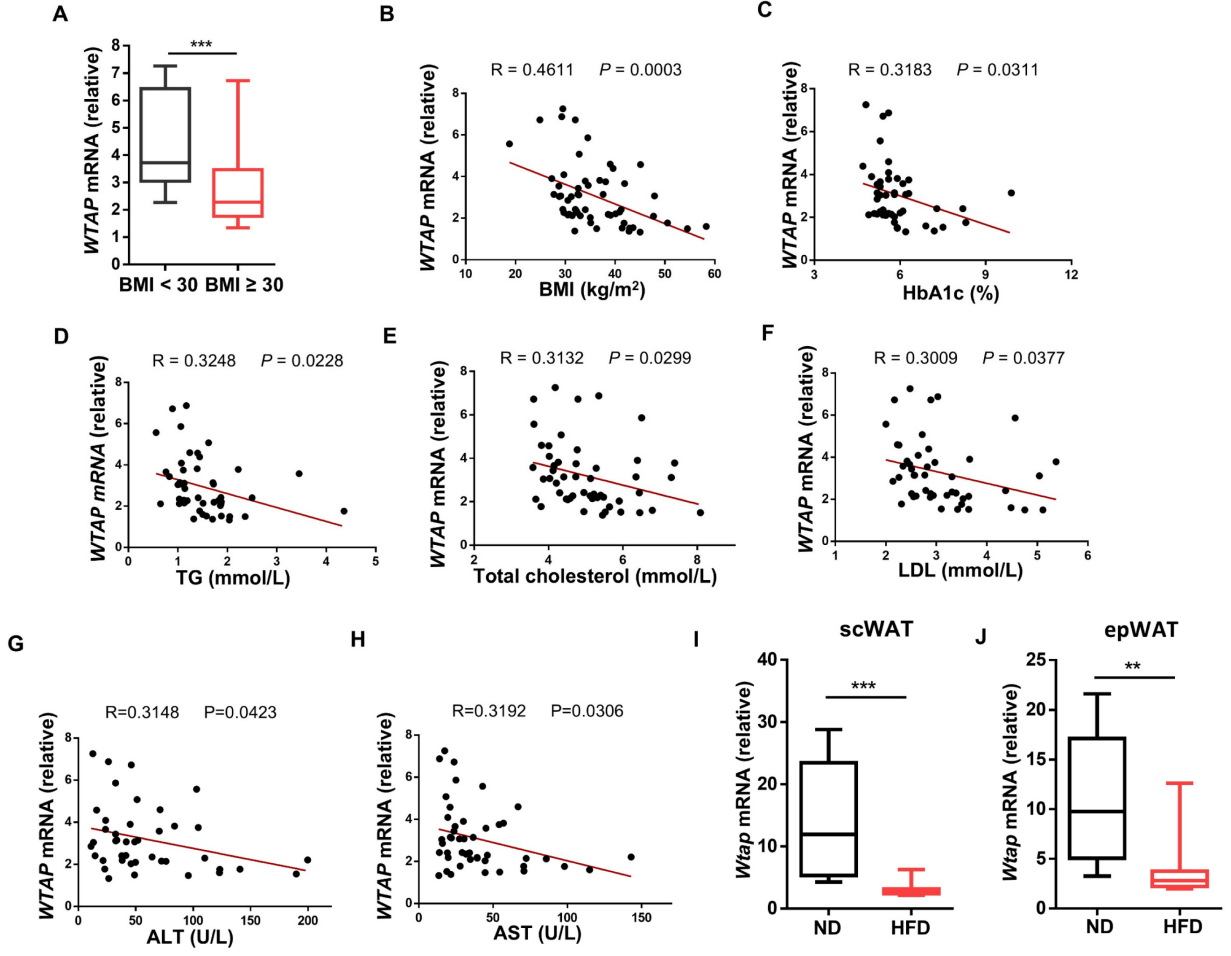
** WTAP expression is decreased in ATMs and negatively related to clinical metabolic traits. A** RT-qPCR analysis of *WTAP* expression in human ATMs from non-obese and obese individuals. BMI < 30: n = 12 individuals; BMI ≥ 30: n = 46 individuals. **B-H** Correlation curves between *WTAP* mRNA expression in human ATMs and clinical metabolic traits: BMI (n = 58), HbA1c (n = 46), TG (n = 49), Total cholesterol (n = 52), LDL (n = 48), ALT (n = 46), and AST (n = 42). **I** *Wtap* mRNA expression in ATMs of scWAT from normal (n = 8) and obese (n = 12) mice. **J** *Wtap* mRNA expression in ATMs of epWAT from normal (n = 12) and obese (n = 12) mice. Data were exhibited as mean ± SEM and analyzed by unpaired Student's *t* test and correlation test. ^**^P < 0.01; ^***^P < 0.001 (A, I-J). Correlation curves were presented by R values and *P* values (B-H).

We first examined WTAP expression in ATMs from non-obese (BMI < 30 kg/m^2^) and obese (BMI ≥ 30 kg/m^2^) subjects. Notably, obese individuals had a lower expression level of *WTAP* in ATMs than non-obese individuals (Fig. 1A). We then assessed the correlation between the levels of *WTAP* and clinical metabolic parameters, and found that *WTAP* transcriptional levels were inversely associated with BMI (Fig. 1B), serum glycosylated hemoglobin (HbA1c) (Fig. 1C), triglyceride (TG) (Fig. 1D), total cholesterol (Fig. 1E), low-density lipoprotein (LDL) (Fig. 1F), ALT (Fig. 1G), and AST levels (Fig. 1H). To confirm this observation, we fed the C57BL/6 mice with normal diet (ND) or HFD for 12 weeks, and isolated ATMs from subcutaneous white adipose tissue (scWAT) and epididymal white adipose tissue (epWAT) for further analysis. Consistent with the expression profiles in humans, significantly lower *Wtap* expression was detected in the ATMs from both scWAT and epWAT of obese mice (Fig. 1I, J). Taken together, these results support that ATMs derived from obese subjects and mice are characterized by the decreased WTAP expressions.

### *Wtap* deficiency attenuates the alternatively activated macrophage program

**Figure 2:**
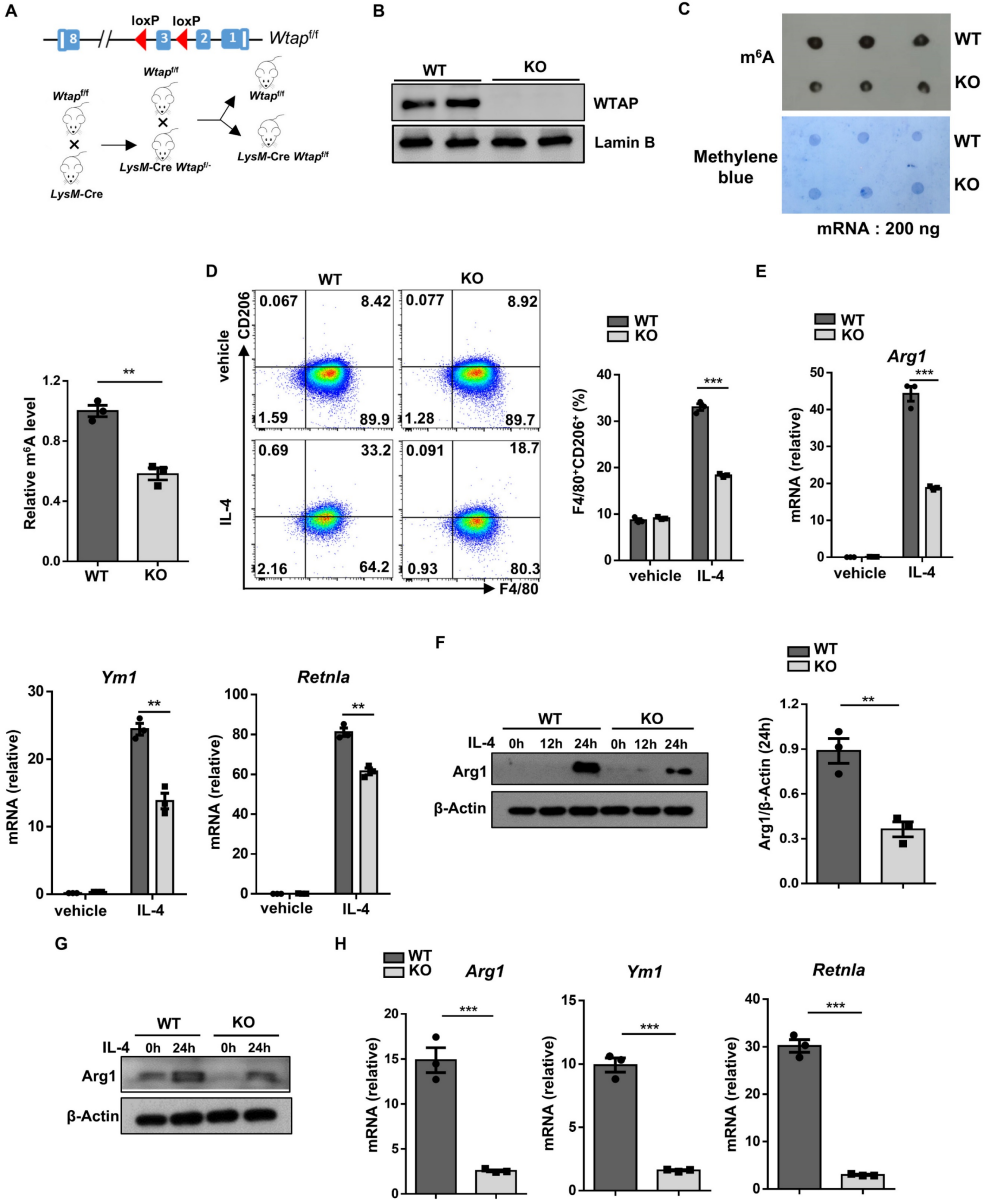
***Wtap* depletion in macrophages downregulates alternative macrophage activation. A** A WTAP-specific knockout mouse model was constructed. LoxP sites were inserted flanking exon 3 of the *Wtap* gene. Crossing these mice with *LysM*-Cre mice resulted in the generation of *LysM*-Cre-*Wtap*^f/f^ mice. **B** Western blot analysis of WTAP protein levels in WT and KO BMDMs. **C** m^6^A dot blot results (top) and methylene blue staining (bottom) in WT and KO BMDMs (200 ng mRNA). Quantitative bar graph was also shown. **D** Representative flow cytometry plots and the percentage of F4/80^+^CD206^+^ macrophages in WT and KO BMDMs with or without IL-4 treatment. **E** RT-qPCR analysis of alternatively activated macrophage-related genes *Arg1*,* Ym1*, and* Retnla* with or without IL-4 stimulation. **F** Arg1 protein expression in IL-4 stimulated BMDMs at 0 h, 12 h and 24 h. **G** Western blot analysis of Arg1 protein levels in peritoneal macrophages with stimulation of IL-4 at 0 h and 24 h. **H** RT-qPCR analysis of *Arg1, Ym1* and* Retnla* expressions in IL-4 stimulated peritoneal macrophages. The results are representative of 3 independent replications (**B** and** F**-**G**). n = 3 for each group (**C**, **E** and **H**). Data were exhibited as mean ± SEM and analyzed by unpaired Student's *t* test.^ *^P < 0.05; ^**^P < 0.01; ^***^P < 0.001.

To elucidate the role of WTAP in the development of obesity, we generated a macrophage-specific *Wtap* knockout mouse model by crossing the *Wtap*^f/f^ mice with the *LysM*-Cre mice (the resulting *LysM*-Cre *Wtap*^f/f^ mice were defined as KO thereafter), and their *Wtap*^f/f^ littermates were served as controls (Fig. 2A). Western blot analysis confirmed that WTAP expression was diminished in bone marrow-derived macrophages (BMDMs) from the KO mice relative to their wild-type (WT) littermates (Fig. 2B), and the m^6^A modification level was significantly decreased in KO BMDMs by dot-blot assays (Fig. 2C). The KO mice survived and grew normally, and displayed similar maturation and activation status of lymphoid cells in the spleen, inguinal lymph nodes (ILNs), and mesenteric lymph nodes (MLNs) (Fig. S1A-D). In addition, the frequencies of dendritic cells (DCs) and macrophages in the bone marrow and spleen from WT and KO mice did not show significant differences, indicating that loss of *Wtap* did not influence the development and maturation of myeloid cells (Fig. S1E, F).

Next, BMDMs were generated from 8-week-old KO mice and WT controls and subjected to IL-4 stimulation. *Wtap* deficiency decreased alternative macrophage polarization (Fig. 2D), and Arg1 expression was downregulated in KO BMDMs as compared to WT BMDMs following IL-4 induction (Fig. 2E, F). Similar results were observed for RT-qPCR analysis of *Ym1* and *Retnla* (Fig. 2E). Consistently, the reduced Arg1 expression was also detected in the KO peritoneal macrophages, as well as *Ym1* and *Retnla* mRNA levels (Fig. 2G, H). As obesity is generally associated with increased circulating palmitate, a saturated fatty acid that could activate TLR-mediated pro-inflammatory signaling pathways 21, we further assessed the effect of *Wtap* deficiency on macrophages in response to palmitate stimulation. Indeed, higher levels of co-stimulatory CD86 expression were noted in KO BMDMs (Fig. S2A). Similarly, an increase of proinflammatory factors was detected in KO BMDMs upon palmitate treatment (Fig. S2B). Furthermore, KO mice demonstrated comparable amounts of total macrophages but CD11c positive ATMs slightly increased in the epWAT as compared to WT controls under normal diet (Fig. S3A-C). Collectively, our data indicate that depletion of *Wtap* represses the alternative macrophage program in BMDMs.

### Loss of *Wtap* exacerbates HFD-induced obesity and systemic metabolic deterioration

**Figure 3:**
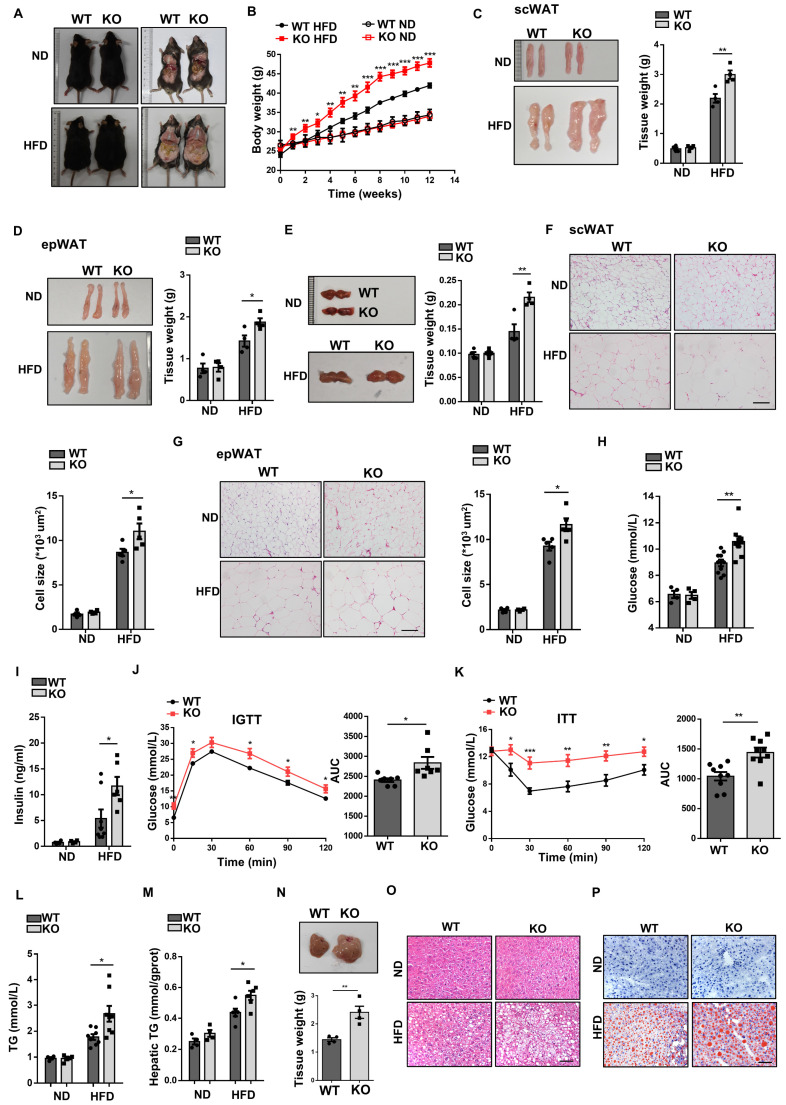
** Macrophage-specific *Wtap* ablation exacerbates HFD-induced obesity and systemic metabolic dysfunction. A** Physical appearance of WT and KO mice after 12 weeks of feeding on a ND or HFD. Male mice were used. **B** Body weight changes in every week under ND or HFD conditions (ND: n = 4; HFD: n = 10). **C-E** Representative images and tissue weights of scWAT (C), epWAT (D) and BAT (E) in WT and KO mice induced by ND or HFD. **F-G** H&E staining of scWAT and epWAT sections and quantified adipocyte areas. Scale bar: 100 μm. **H** Random blood glucose levels in WT and KO mice fed with ND or HFD. **I** Plasma insulin levels in WT and KO mice induced by ND or HFD. **J-K** Results of intraperitoneal glucose tolerance test (IGTT) and insulin resistance test (ITT) under HFD, including areas under the curves (AUC) analysis. **L-M** Triglyceride concentrations in plasma (L) and hepatic tissues (M) from WT and KO obese mice.** N** Representative images and tissue weights of the liver following HFD feeding in WT and KO mice. **O-P** H&E staining and Oil Red O staining on liver sections from WT and KO mice fed a ND or induced with HFD. Scale bar: 50 μm. Data were presented as mean ± SEM and analyzed by unpaired Student's *t* test.^ *^P < 0.05; ^**^P < 0.01; ^***^P < 0.001.

Given the crucial role of macrophages played in the development of obesity, the KO mice and WT littermates were fed with either ND or HFD for 12 weeks. Under ND, no perceptible phenotypical difference was observed between two groups of mice. However, KO mice gained more body weight as compared to WT mice after HFD feeding (Fig. 3A, B), which was correlated with a significant increase of fat-pad weight, including scWAT (Fig. 3C), epWAT (Fig. 3D), and BAT (Fig. 3E). In line with increased fat mass, histological analysis showed larger adipocytes in the WATs of KO mice (Fig. 3F, G). We also observed higher random blood glucose levels and hyperinsulinemia in KO mice (Fig. 3H, I), along with a significant impairment in glucose tolerance (Fig. 3J) and insulin sensitivity (Fig. 3K), although there was no difference between those mice under normal diet (Fig. S4A, B). In addition, we examined the impact of *Wtap* deficiency on lipid metabolism. The KO mice showed markedly increased plasma and hepatic triglycerides (Fig. 3L, M), higher liver weights (Fig. 3N), as well as higher intrahepatic lipid accumulation as determined by H&E and Oil Red O staining (Fig. 3O, P). Altogether, our results suggest that macrophage-specific *Wtap* ablation promotes the development of obesity and obesity-associated metabolic deterioration.

### *Wtap* is required to maintain adipose immune homeostasis in HFD-induced mice

**Figure 4:**
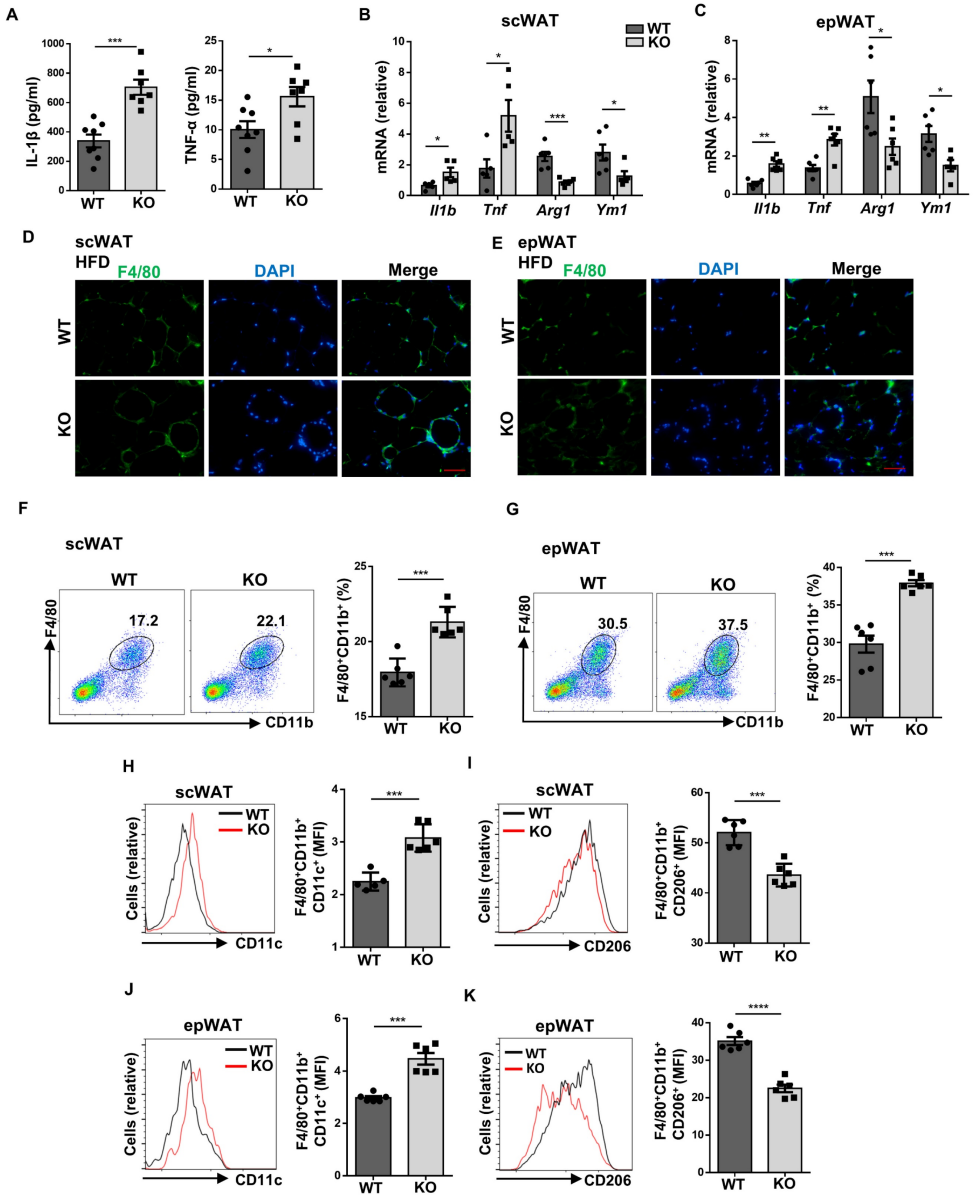
***Wtap* deficiency causes imbalance of ATMs. A** ELISA results of inflammatory cytokines IL-1β and TNF-α in plasma of WT and KO mice under HFD (n = 7). **B-C** RT-qPCR analysis of presented genes in scWAT and epWAT from WT and KO mice after HFD (n = 5). **D-E** Immunofluorescence images of F4/80^+^ crown-like structures in scWAT and epWAT sections. Scale bar: 50 μm. **F-G** Representative flow cytometry plots and the analysis of total F4/80^+^CD11b^+^ macrophages in scWAT and epWAT (n = 6). **H-K** Expression of CD11c and CD206 in F4/80^+^CD11b^+^ macrophages as shown (H, J). The amounts of F4/80^+^CD11b^+^CD11c^+^ and F4/80^+^CD11b^+^CD206^+^ macrophages were quantified and shown as relative mean fluorescence intensity (MFI) (I, K). Data were presented as mean ± SEM and analyzed by unpaired Student's *t* test.^ *^P < 0.05; ^**^P < 0.01; ^***^P < 0.001.

Next, we sought to investigate the effect of *Wtap* depletion on HFD-induced immune disorders. The levels of pro-inflammatory cytokines IL-1β and TNF-α were significantly higher in the serum of KO mice following HFD challenge (Fig. 4A). Similarly, upregulated *Il1b* and *Tnf*, but downregulated *Arg1* and *Ym1* mRNA levels were detected in the WATs from HFD-fed KO mice (Fig. 4B, C). HFD significantly enhanced the formation of F4/80^+^ CLSs in the WATs from KO mice as evidenced by the immunostaining (Fig. 4D, E). Flow cytometry analysis of WATs further validated the increased infiltration of macrophages in the KO mice (Fig. 4F, G). Moreover, the KO mice manifested a remarkable reduction of alternatively activated macrophages coupled with an increase of classical activated phenotype both in the scWAT (Fig. 4H, I) and epWAT (Fig. 4J, K). Therefore, *Wtap* deficiency in macrophages aggravates HFD-induced immune imbalance.

### Loss of *Wtap* suppresses energy expenditure and WAT beiging process

**Figure 5:**
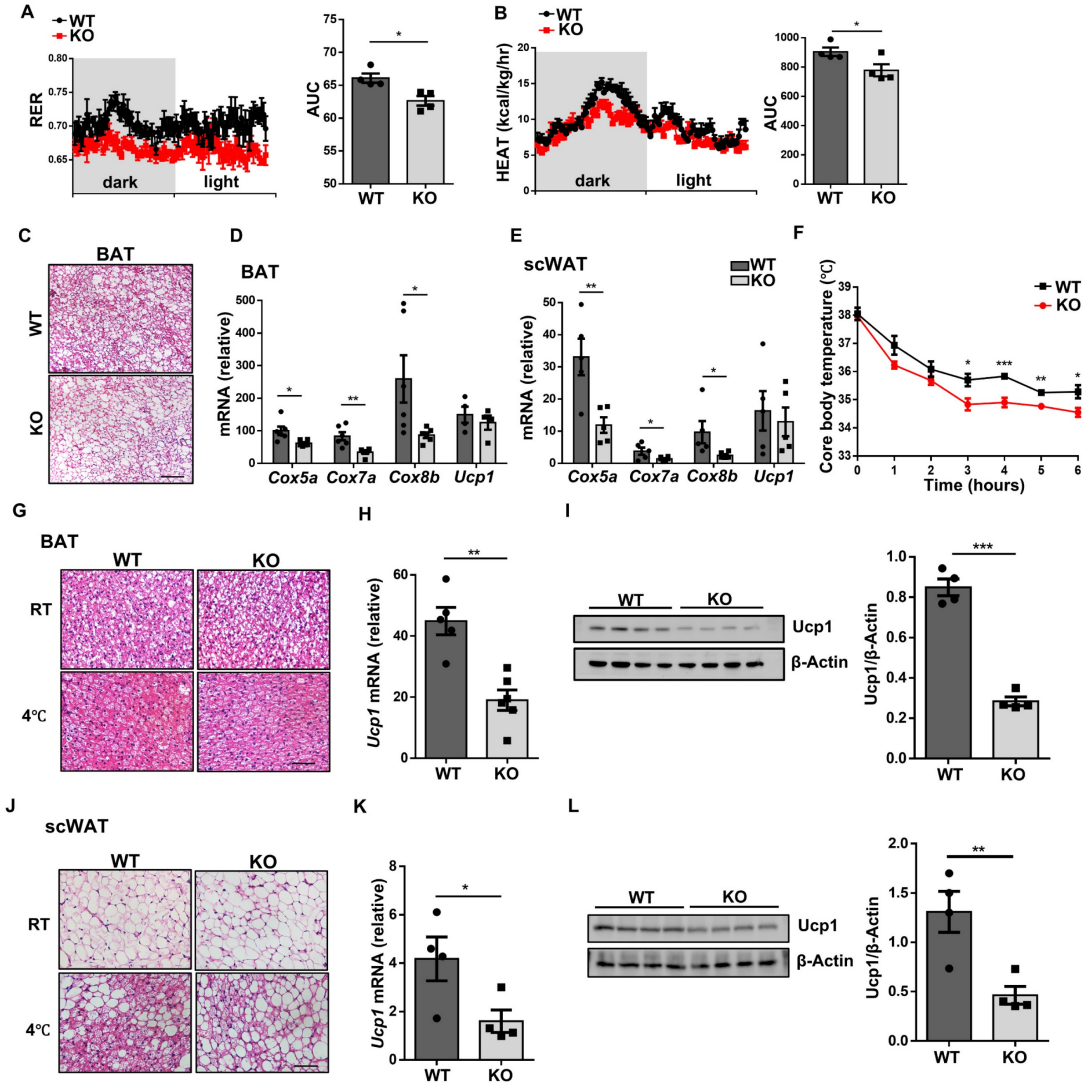
** Loss of *Wtap* downregulates energy expenditure and adipose tissue beiging. A-B** Real-time monitoring of RER and heat production in HFD over 24 h for HFD mice, including areas under the curves analysis. **C** Representative H&E staining images of BAT sections from WT and KO mice with HFD. Scale bar: 100 μm. **D-E** RT-qPCR results of indicated thermogenesis genes in scWAT and BAT from HFD mice.** F** Core body temperature records during the initial 6 h of exposure to 4 °C environment (n = 6). **G** Representative H&E staining images of BAT sections following room temperature or cold stimulation. Scale bar: 50 μm. **H-I** RT-qPCR and western blot analysis of Ucp1 expression in BAT from WT and KO mice exposed to 4 °C. **J** Representative H&E staining images of scWAT sections under room temperature or cold condition. Scale bar: 50 μm. **K-L** RT-qPCR and western blot analysis of Ucp1 expression in scWAT from WT and KO mice with cold stimulation. Male mice were used in cold stimulation mouse model. n = 4 for each group. (**I, L**) Data were presented as mean ± SEM and analyzed by unpaired Student's *t* test.^ *^P < 0.05; ^**^P < 0.01; ^***^P < 0.001.

Given that ATMs have been well recognized to play a critical role during thermogenesis 22, we thus next examined whether *Wtap* deficiency in macrophages impacts energy homeostasis. Metabolic assays revealed that HFD challenge rendered the KO mice with significantly lower respiratory exchange ratio (RER) and heat production as compared to that of WT mice (Fig. 5A, B), although they exhibited comparable RER (Fig. S4C) and heat production (Fig. S4D) as control mice once they were under ND condition. Since BAT is the major adipose tissue to dissipate energy, we therefore assumed that the reduced heat production might be attributed to the decline of BAT metabolic activity in the KO mice. Indeed, much higher lipid-droplet content was observed in the BAT of KO mice upon the challenge of HFD (Fig. 5C). Consistently, the KO mice were featured by the decreased expression of the vital thermogenic genes in the BAT, including *Cox5a*, *Cox7a*, and *Cox8b* (Fig. 5D). Notably, similar results were also observed in scWAT (Fig. 5E), suggesting that the beiging process is likely implicated in the repressed energy expenditure conferred by *Wtap* deficiency as well.

To validate the above assumption, randomly selected KO and WT mice were subjected to cold stimulation (4 °C) as described. It was found that the core body temperatures were lower in KO mice (Fig. 5F), and the KO mice manifested attenuated BAT activity as evidenced by the increased BAT lipid-droplet levels (Fig. 5G) coupled with repressed Ucp1 expressions (Fig. 5H, I). H&E staining of the scWAT sections revealed the formation of multilocular beige adipocytes in WT mice after cold exposure, but it was markedly decreased in the KO mice (Fig. 5J), and scWAT from KO mice displayed a markedly lower Ucp1 expression at both transcriptional and protein levels (Fig. 5K, L). Together, these results demonstrate that *Wtap*-deficient ATMs limit BAT activity and WAT beiging process during adaptive thermogenesis.

### WTAP enhances *Idh1* mRNA stability and translation by mediating its m^6^A modification

**Figure 6:**
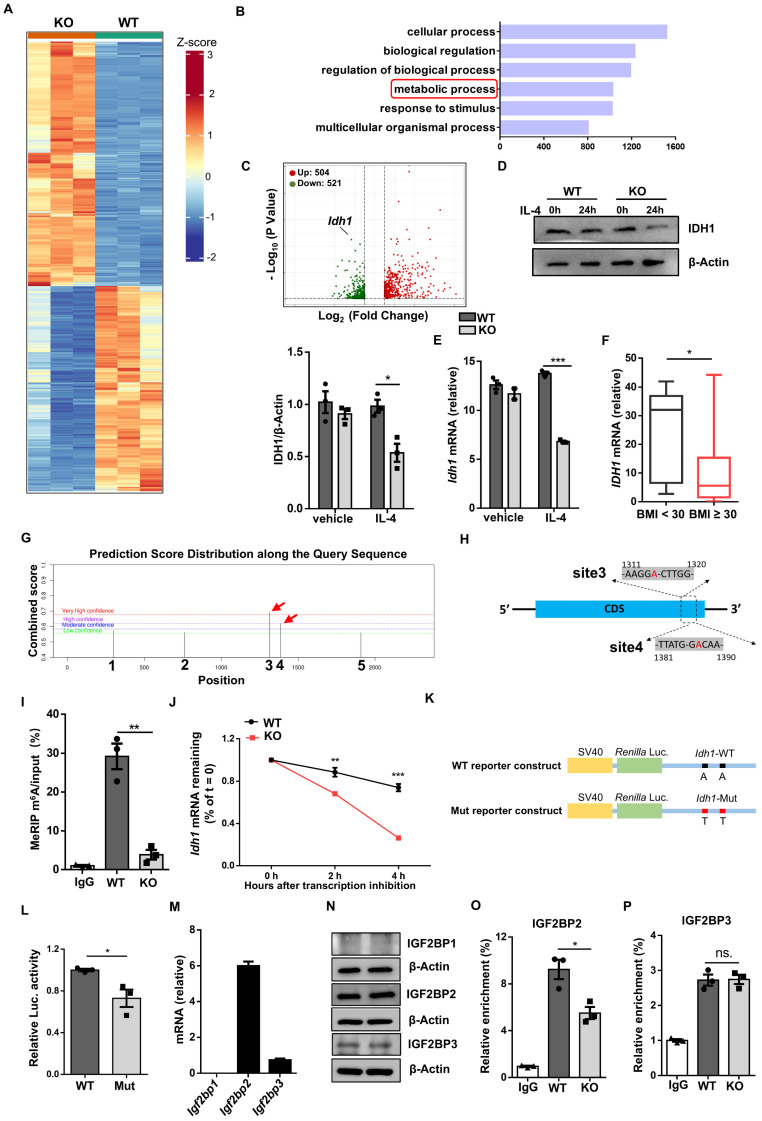
***Wtap* deficiency decreases *Idh1* m^6^A modification and impairs the mRNA stability and translation efficacy. A** Heatmap depicting gene expression differences between WT and KO BMDMs. Three biological replicates for each group were included. **B** GO enrichment analysis of the top six biological process pathways. The abscissa represents the number of enriched genes. **C** Volcano plot of differential gene expression in metabolic process. **D-E** RT-qPCR and western blot results of Idh1 expression in WT and KO BMDMs following IL-4 treatment. **F** RT-qPCR analysis of *IDH1* expression in human ATMs from non-obese and obese individuals. BMI < 30: n = 9 individuals; BMI ≥ 30: n = 33 individuals **G** Five potential predicted *Idh1* mRNA modification sites, along with their predicted scores distribution. Red arrows indicate the top-ranked two sites based on the scores. **H** Schematic representative positions of m^6^A modification within the transcripts of *Idh1*. **I** Abundance of methylated *Idh1* transcripts measured in mRNA samples immunoprecipitated with anti-m^6^A antibody or IgG in WT and KO BMDMs.** J** RT-qPCR analysis of *Idh1* mRNA remaining levels in BMDMs following treatment with 5 μg/ml Actinomycin D for 0 h, 2 h and 4 h. **K** Wild-type or mutated m^6^A sequences (A to T mutations) of *Idh1* in conjunction with the Renilla luciferase reporter in psiCHECK^TM^-2 vector. **L** Relative luciferase activities of HEK293T cells that were transfected with WT or mutant plasmid, and normalized to firefly luciferase activities. **M-N** RT-qPCR and western blot analysis of IGF2BP1, IGF2BP2 and IGF2BP3 expression in BMDMs. **O-P** RIP-qPCR analysis of the relative enrichment of *Idh1* mRNA combined with IGF2BP2 and IGF2BP3 in WT and KO BMDMs. Results are representative of 3 independent replications (**D** and **N**), n = 3 for each group (**E**, **I**-**J**, **L**-**M** and **O**-**P**) Data were presented as mean ± SEM and analyzed by unpaired Student's *t* test.^ *^P < 0.05; ^**^P < 0.01; ^***^P < 0.001.

To gain insights into the molecular mechanisms by which WTAP modulates ATM program, deep RNA sequencing (RNA-seq) was conducted. As compared to WT BMDMs, the KO BMDMs displayed 1,097 upregulated and 919 downregulated genes (Fig. 6A). Gene ontology (GO) analysis revealed that the differentially expressed genes (DEGs) were enriched in metabolic process (Fig. 6B), among which *Idh1* was the most downregulated one, and was then selected for the follow up studies (Fig. 6C). Indeed, RT-qPCR and Western blot confirmed that KO BMDMs were characterized by the attenuated IDH1 expression following IL-4 induction (Fig. 6D, E), but no discernable difference was observed under unstimulated condition. Meanwhile, ATMs from obese individuals had decreased expression of *IDH1* as compared to non-obese individuals (Fig. 6F). SRAMP (sequence-based RNA adenosine methylation site predictor) predicted 5 possible m^6^A modification sites in the *Idh1* transcript, with high confidence at sites 3 and 4 (Fig. 6G, H). Therefore, we designed primers covering these two sites to detect the change of m^6^A levels. Methylated RNA immunoprecipitation (MeRIP)-qPCR analysis revealed that the *Idh1* transcripts were effectively enriched by m^6^A-specific antibody, and m^6^A modification of *Idh1* was remarkably decreased in *Wtap* deficient BMDMs (Fig. 6I).

Given that one of the critical features of m^6^A modification is to regulate mRNA degradation 23, we thus first examined whether WTAP-mediated m^6^A is involved in *Idh1* mRNA stability. Excitingly, RNA decay assays showed that *Wtap* deficiency significantly decreased the half-life of *Idh1* mRNA in BMDMs (Fig. 6J). We then embarked on whether WTAP promotes IDH1 expression at the translational level. For this purpose, we mutated the putative m^6^A modified adenosines (A) to thymines (T) in the *Idh1* mRNA and constructed a reporter plasmid (Fig. 6K). Luciferase reporter assay showed that the luciferase activity of the mutant plasmid was significantly lower than that of WT construct (Fig. 6L), indicating a decreased translation efficiency.

As IGF2BP proteins act as m^6^A readers to enhance the stability and translation of targeted transcripts 19, we next wondered whether they were associated with the alteration of m^6^A-modified *Idh1*. Since IGF2BP1 was almost undetectable in the BMDMs, we thus focused on IGF2BP2/3 (Fig. 6M, N). RIP-qPCR analysis revealed that both IGF2BP2 and IGF2BP3 bound to the regions flanking the above indicated m^6^A sites, but only IGF2BP2 was associated with *Idh1* mRNA less efficiently in KO BMDMs (Fig. 6O, P), suggesting that IGF2BP2 might contribute to m^6^A-mediated enhancement of *Idh1* mRNA stability and translation. To further verify the critical role of IGF2BP2 in these processes, we silenced *Igf2bp2* expression in BMDMs (Fig. S5A-B). Notably, the reduced mRNA stability of *Idh1* observed in KO BMDMs was effectively rescued upon *Igf2bp2* knockdown (Fig. S5C). Meanwhile, when HEK293T cells were co-transfected with either wild-type or mutant plasmids along with *IGF2BP2* siRNA, *IDH1* translation efficiency exhibited no difference, as demonstrated by the luciferase reporter assay (Fig. S5D-F). Collectively, these results demonstrate that WTAP-mediated m^6^A regulates *Idh1* mRNA stability and translation efficiency to promote its expression.

### *Wtap* promotes IDH1-catalyzed α-KG generation to modulate macrophage program

**Figure 7:**
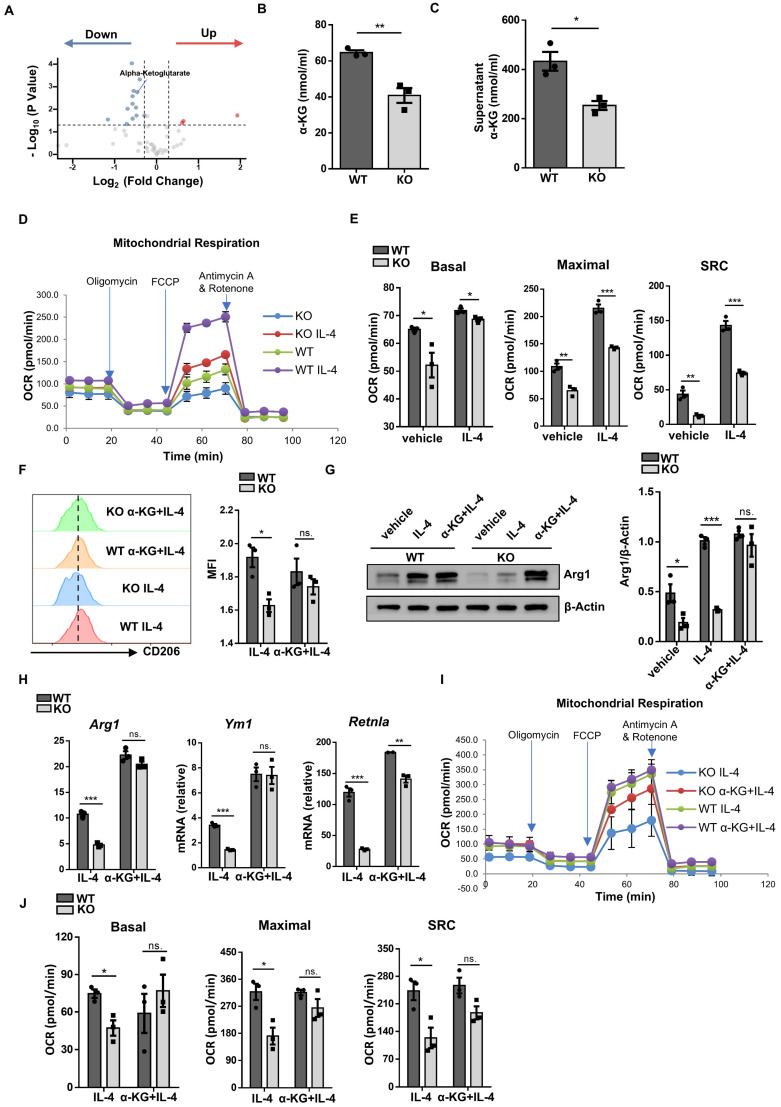
***Wtap* promotes IDH1-catalyzed α-KG generation to modulate macrophage plasticity. A** Scatter plot of differential metabolites from metabolomics analysis, with each point representing a single metabolite. Four biological replicates for each group were included**. B-C** Alpha-KG concentrations in cells and culture supernatants of WT and KO BMDMs. **D** Oxygen consumption rate (OCR) of BMDMs from WT and KO mice stimulated by IL-4 with treatment of oligomycin, FCCP, rotenone, and antimycin A. **E** Quantitative statistics of basal mitochondria OCR, maximal respiration capacity and spare respiratory capacity (SRC) in WT and KO BMDMs. **F** Expression of CD206 in F4/80^+^ macrophages in WT and KO BMDMs stimulated by IL-4 with or without supplementation of α-KG. **G** Western blot analysis of Arg1 protein levels in BMDMs with indicated treatment groups. **H** RT-qPCR analysis of *Arg1, Ym1* and *Retnla* expression in BMDMs under indicated conditions. **I** OCR of IL-4 stimulated BMDMs from WT and KO mice with addition of α-KG or not under treatment of oligomycin, FCCP, rotenone, and antimycin A. **J** Quantitative analysis of basal OCR, maximal OCR and SRC in WT and KO BMDMs. n = 3 for each group (**B**-**E** and **H**-**J**). Results are representative of 3 independent replications (**F**). Alpha-KG: 100 nmol/ml (**F**-**J**). Data were presented as mean ± SEM and analyzed by unpaired Student's *t* test. ns. no significance; ^*^P < 0.05; ^**^P < 0.01; ^***^P < 0.001.

As GO analysis of RNA-seq data indicated that loss of *Wtap* resulted in altered pathways relevant to metabolic process, we performed metabolomic assays using WT and KO BMDMs. Consistent with reduced IDH1 expression, a metabolic enzyme responsible for converting isocitrate to α-KG, KO BMDMs were featured by the reduced α-KG levels (Fig. 7A). Although isocitrate and glutamate are both crucial for α-KG production (Fig. S6A) 24, DEGs revealed that other crucial enzymes in generating α-KG were hardly affected between WT and KO BMDMs (Fig. S6B), which were consistent with the RT-qPCR results (Fig. S6C). Alpha-KG assay further confirmed that *Wtap* deficiency decreased the levels of α-KG both in the cells and culture supernatants (Fig. 7B, C). Moreover, there is feasible evidence that α-KG, as a metabolic regulator, augments fatty acid oxidation (FAO) 25, which would be in favor of alternatively activated macrophage program. We thus next compared oxygen consumption rate (OCR) between WT and KO macrophages. The KO BMDMs displayed markedly lower basal mitochondrial OCR, maximal respiration capability, and spare respiratory capacity (SRC) following IL-4 induction (Fig. 7D, E), indicating a decreased commitment to oxidative phosphorylation (OXPHOS).

Finally, a rescue experiment was performed by adding α-KG into the BMDM culture supernatants. Supplement of α-KG completely restored the expression of CD206 (Fig. 7F), Arg1 (Fig. 7G, H), and other alternatively activated macrophage markers (Fig. 7H). Consistently, in the presence of α-KG, KO BMDMs manifested comparative basal mitochondrial OCR, maximal respiration capability, and SRC as that of WT BMDMs (Fig. 7I, J). Taken together, these data support that *Wtap* depletion downregulates IDH1-catalyzed α-KG production, thereby impacting OXPHOS to attenuate IL-4 stimulated macrophage program.

## Discussion

m^6^A is the most prevalent mRNA modification in eukaryotes. Studies on m^6^A indicate that it has a vital impact on a variety of physiological and pathological processes, including stem-cell pluripotency, brain function, immune response and tumorigenesis 23. Of note, studies also suggested that METTL3 promotes macrophage activation by modulating STAT1 26 and IRKAM signaling 27. However, controversial results were also reported with different stimulation and status. For example, METTL3 was noted capable of inhibiting macrophage M2 program as well once it targets on Trib1/ERK/STAT3 signaling 28. Given the critical role of macrophages in the maintenance of immune homeostasis in adipose tissues, the exact impact of m^6^A on adipose macrophages, particularly on their program under obese settings, warrants further investigations. In the present report, we provided convincing evidence demonstrating a role of WTAP-mediated m^6^A modification in the maintenance of macrophage homeostasis, in which loss of *Wtap* in macrophages exacerbated HFD-induced obesity.

In general, epitranscriptomic factors and metabolic pathways contribute to the regulation of macrophage polarization and functional plasticity 29, while the mechanisms by which macrophages integrate these intricate networks are still largely unknown. As a regulatory subunit of RNA methyltransferase complex, WTAP-mediated m^6^A modification constitutes an epitranscriptomic layer of regulation to guarantee the macrophage program. Our RNA-seq data revealed significant changes of many metabolism-related genes in *Wtap* deficient macrophages. Among those, *Idh1* was the most significantly downregulated gene. IDH1 is a crucial enzyme for the tricarboxylic acid (TCA) cycle and catalyzes oxidative decarboxylation of isocitrate to α-KG. Using MeRIP-qPCR, we confirmed that *Idh1* mRNA is the target of WTAP-mediated m^6^A modification. Loss of *Wtap* reduced *Idh1* mRNA stability and its translation efficiency, thereby contributing to the decline of its mRNA and protein expression levels. The recognition and interpretation of m^6^A modification rely on its reader proteins, YTHDFs and IGF2BPs. The binding of YTHDFs leads to the localization of their target mRNAs from the translatable pool to cellular RNA decay sites 30. Therefore, mRNA levels tend to be upregulated when m^6^A abundance is decreased in the YTHDF2-mediated decay pathway. In contrast to the mRNA decay-promoting function of YTHDFs, IGF2BPs preferentially enhance the stability and translation of bound mRNA 19. In line with these previous reports, the consensus GG(m^6^A)C sequence-containing regions in the *Idh1* transcript were highly bound by IGF2BP2 that decreased remarkably upon *Wtap* ablation, indicating a role of IGF2BP2 in promoting the stability and translation of *Idh1* mRNA.

Metabolomic profiling showed that *Wtap* deficiency changed the levels of several important metabolic intermediates in macrophages. TCA cycle intermediate α-KG was one of the most significantly downregulated metabolites in macrophages deficient in *Wtap*, which was in line with the reduced expression of IDH1. Alpha-KG was previously demonstrated to be a cofactor of the JMJD3 histone demethylase that targets H3K27me3 25. Moreover, it is important to note that α-KG serves as a metabolic regulator that governs the engagement of OXPHOS. Notably, accumulated evidence indicates that macrophages with an alternatively activated phenotype possess an intact TCA cycle, and prefer oxidative metabolism, particularly FAO, to support their energy demand 31, 32. Indeed, *Wtap* deficient macrophages exhibited significantly lower OXPHOS than those of WT macrophages in the presence of IL-4, as evidenced by the decreased OCR. Consistently, α-KG supplementation almost completely rescued the alteration of oxidative metabolism, supporting that *Wtap* deficiency impairs macrophage program at least in part by hampering IDH1-regulated α-KG production.

Previous studies also suggested a critical role for α-KG in adipocyte activity. Specifically, α-KG was noted capable of facilitating TET-mediated DNA demethylation in the *Prdm16* promoter, which is required for the progenitor commitment to brown adipocyte differentiation 33. Alpha-KG not only increases the expression of thermogenic genes (i.e., *Ucp1*, *Dio2*, and *Cidea*) in the BAT, but also enhances lipolysis in the WATs through OXGR1-dependent adrenal activation, and as a result, administration of exogenous α-KG prevents HFD-induced obesity in mice 34. In line with these observations, human plasma α-KG levels are inversely associated with several metabolic risk factors, including body weight, BMI, waist circumference, and hip circumference 35. These published results, together with our data, support that α-KG could be a viable metabolite against obesity in clinical settings. However, population-based studies would be necessary to prove the clinical benefits.

It is worthy of note, we failed to detect a significant difference between WT and KO mice under normal diet, while significant alterations were observed upon HFD challenge. This discrepant outcome suggests that the functional role of WTAP may become critical under metabolic stress. Similar as DNA methylation, RNA methylation levels would not change under physiological conditions, while HFD-induced metabolic stress would be coupled with an RNA methylation turnover as manifested by the change of RNA methylation levels and/or patterns 36. WTAP acts as a critical cofactor for RNA methylation, its deficiency would significantly impair the HFD-induced RNA methylation turnover, which would, therefore, lead to the presence of related metabolic phenotypes.

In summary, we demonstrated convincing evidence that ATMs originating from obese subjects are characterized by the reduced WTAP expression. Studies in animal models illustrated that loss of *Wtap* restricts alternatively activated macrophage program. As a result, mice with macrophages deficient in *Wtap* manifest aggravated obesity following HFD challenge. Mechanistically, WTAP-mediated m^6^A of *Idh1* mRNA promotes its stability and translation efficiency, thereby increasing isocitrate catabolism to produce α-KG. Alpha-KG further modulates metabolic reprogramming essential for macrophage polarization. Our results provide insights into the role of WTAP-mediated m^6^A modification in macrophage functional plasticity, which may help to identify novel therapeutic targets against obesity and other metabolic diseases in clinical settings.

## Supplementary Material

Supplementary figures and tables.

## Abbreviations

m^6^A: *N*^6^-methyladenosine
WTAP: Wilms tumor 1-associated protein
IDH1: isocitrate dehydrogenase 1
ATMs: adipose tissue macrophages
scWAT: subcutaneous white adipose tissue
epWAT: epididymal white adipose tissue
BMDM: bone marrow derived macrophage
HFD: high-fat diet
ND: normal diet
BAT: brown adipose tissue
CLSs: crown-like structures
RER: respiratory exchange ratio
Ucp1: Uncoupling protein1
MeRIP: methylated RNA immunoprecipitation
IGF2BPs: insulin-like growth factor 2 mRNA binding proteins
DEGs: differentially expressed genes
OCR: oxygen consumption rate
SRC: spare respiratory capacity
OXPHOS: oxidative phosphorylation
α-KG: α-ketoglutarate
TCA cycle: tricarboxylic acid cycle
