# Supplementary material for: WTAP Maintains Alternative Activation of Macrophages by Promoting IDH1-Mediated α-ketoglutarate Production
Source: Int J Biol Sci. 2025 Aug 22;21(12):5428–43. doi: 10.7150/ijbs.115672 (PMC12435483; doi:10.7150/ijbs.115672)
Supplement: Supplementary file 1 — Supplementary figures and tables. [file ijbsv21p5428s1.pdf]

Supplementary Materials for

WTAP maintains macrophage homeostasis to attenuate HFD-induced obesity by promoting IDH1-mediated α-ketoglutarate production

Qianqian Xu[1,†], Jing Zhang[1,†], Yuan Zou[2,†], Longmin Chen[2], Fei Sun[1], Xi Luo[1], Ting Wang[1], Yang Li[1], Shu Zhang[1], Fei Xiong[1], Qilin Yu[1], Ping Yang[1], Quan Gong[3], Shi-Wei Liu[4,*] and Cong-Yi Wang[4,5,6*]

[1]Department of Respiratory and Critical Care Medicine, the Center for Biomedical Research, NHC Key Laboratory for Respiratory Diseases, Tongji Hospital, Tongji Medical College, Huazhong University of Science and Technology, Wuhan, China.

[2]Department of Rehabilitation, Tongji Hospital, Tongji Medical College, Huazhong University of Science and Technology, Wuhan, China.

[3] Department of Immunology, School of Medicine, Yangtze University, Jingzhou, China.

[4]Shanxi Bethune Hospital, Shanxi Academy of Medical Science, Tongji Shanxi Hospital, Third Hospital of Shanxi Medical University, the Key Laboratory of Endocrine and Metabolic Diseases of Shanxi Province, Taiyuan, China.

[5]The Center for Biomedical Research, Tongji Hospital Research Building, Tongji Hospital, Tongji Medical College, Huazhong University of Science and Technology, Wuhan, China.

[6]Diabetes Research Center, Qatar Biomedical Research Institute, Hamad Bin Khalifa University, Doha, Qatar.

[†]These authors contributed equally to this work.

[*]All correspondence should be addressed to Drs. Cong-Yi Wang (wangcy@tjh.Tjmu.edu.cn or cwang@hbku.edu.qa) and Shi-Wei Liu

(lswspring6@aliyun.com).

The file includes:

Fig. S1. Depletion of *Wtap* has no effects on either T cell activation or myeloid cell development and maturation.

Fig. S2. *Wtap* deletion promotes macrophage activation in response to palmitate stimulation.

Fig. S3. The effects of *Wtap* deficiency on ATMs under ND.

Fig. S4. KO mice present comparable glucose tolerance, insulin sensitivity, RER and heat production with WT mice fed with ND.

Fig. S5. IGF2BP2 modulates *Idh1* mRNA stability and translation.

Fig. S6. Expression profile of the genes critical for α-KG production.

Table S1. Clinical characteristics of subjects

Table S2. Primer sequences for RT-qPCR

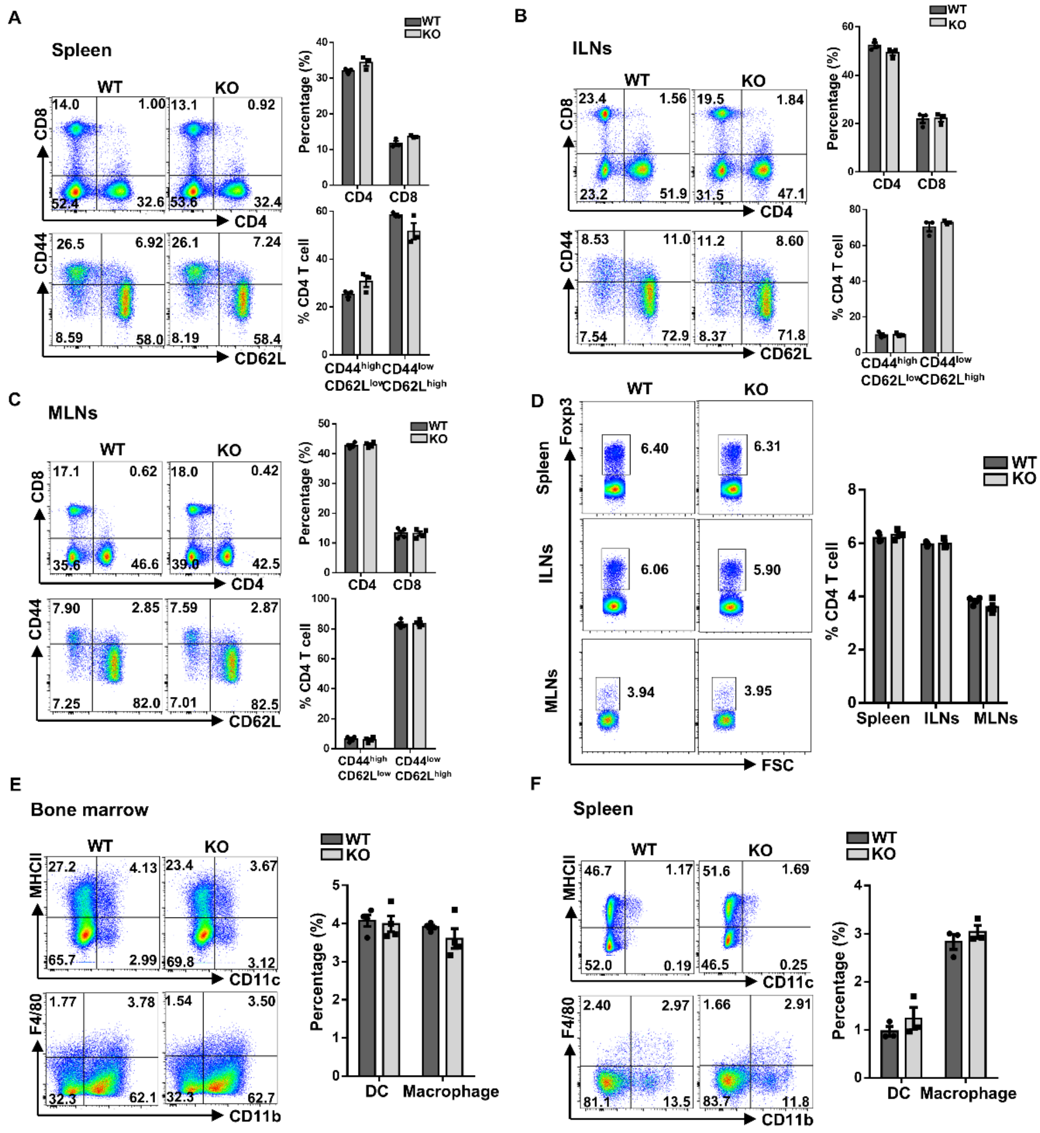

Fig. S1. Depletion of *Wtap* has no effects on either T cell activation or myeloid cell development and maturation. A-C Representative FACS proportion of $CD4^+$, $CD8^+$, $CD4^+$ effector memory ($CD44^{high}CD62L^{low}$) and naive ($CD44^{low}CD62L^{high}$) T cells in the spleen (n = 3), inguinal lymph nodes (ILN) (n = 3) and mesenteric lymph nodes (MLN) (n = 4) from WT and KO mice. **D** Representative FACS proportion of $Foxp3^+$ Tregs gated from $CD4^+$ T cells in spleen, ILN and MLN. **E-F** Representative FACS proportion and percentage of $CD11c^+MHCII^+$ DCs and $F4/80^+CD11b^+$ macrophages from bone marrow (E) and spleen (F). Data were exhibited as mean ± SEM and analyzed by unpaired Student's *t* test. All results presented no significant differences.

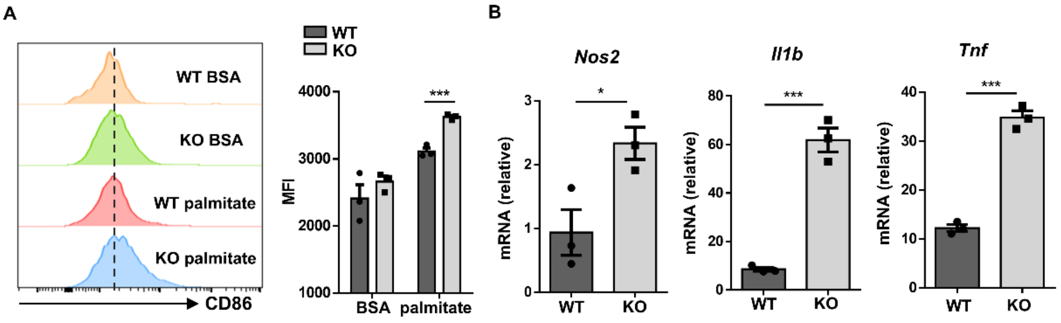

Fig. S2. *Wtap* deletion promotes macrophage activation in response to palmitate stimulation. A-B Expression of CD86 and quantified MFI in F4/80$^+$ macrophages under treatment of BSA or palmitate. **C** RT-qPCR analysis of *Nos2, Il1b and Tnf* in WT and KO BMDMs with addition of palmitate. Data were exhibited as mean ± SEM and analyzed by unpaired Student's *t* test. $^*P < 0.05$; $^{***}P < 0.001$.

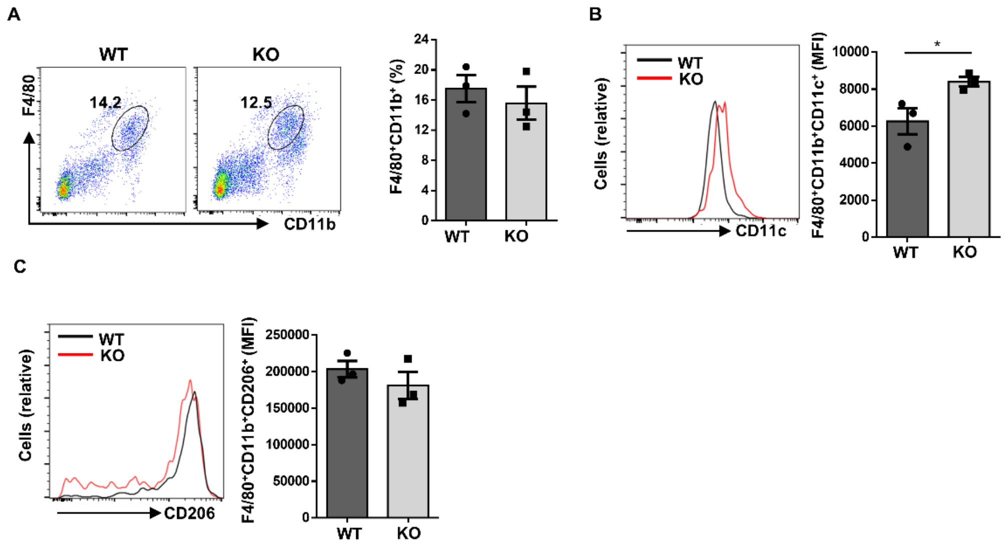

Fig. S3. The effects of *Wtap* deficiency on ATMs under ND. A Representative frequency plots of total macrophages in epWAT from WT and KO mice (n = 3) **B-C** Expression of CD11c and CD206 in F4/80$^+$CD11b$^+$ macrophages from WT and KO mice. Data were exhibited as mean ± SEM and analyzed by unpaired Student's *t* test. $^*P < 0.05$.

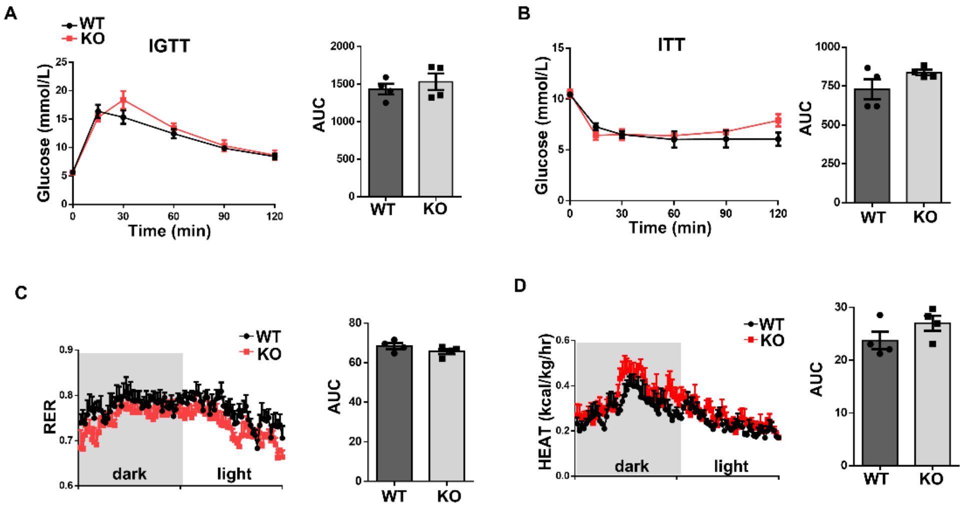

Fig. S4. KO mice present comparable glucose tolerance, insulin sensitivity, RER and heat production with WT mice fed with ND. A-B The results of intraperitoneal glucose tolerance test (IGTT) (I) and insulin resistance test (ITT), and the analysis of areas under curves. **C-D** The monitoring of real-time RER and heat production of ND mice for 24 h, and the areas under the curves were quantitatively analyzed. Data were exhibited as mean ± SEM and analyzed by unpaired Student's *t* test. All results presented no significant differences.

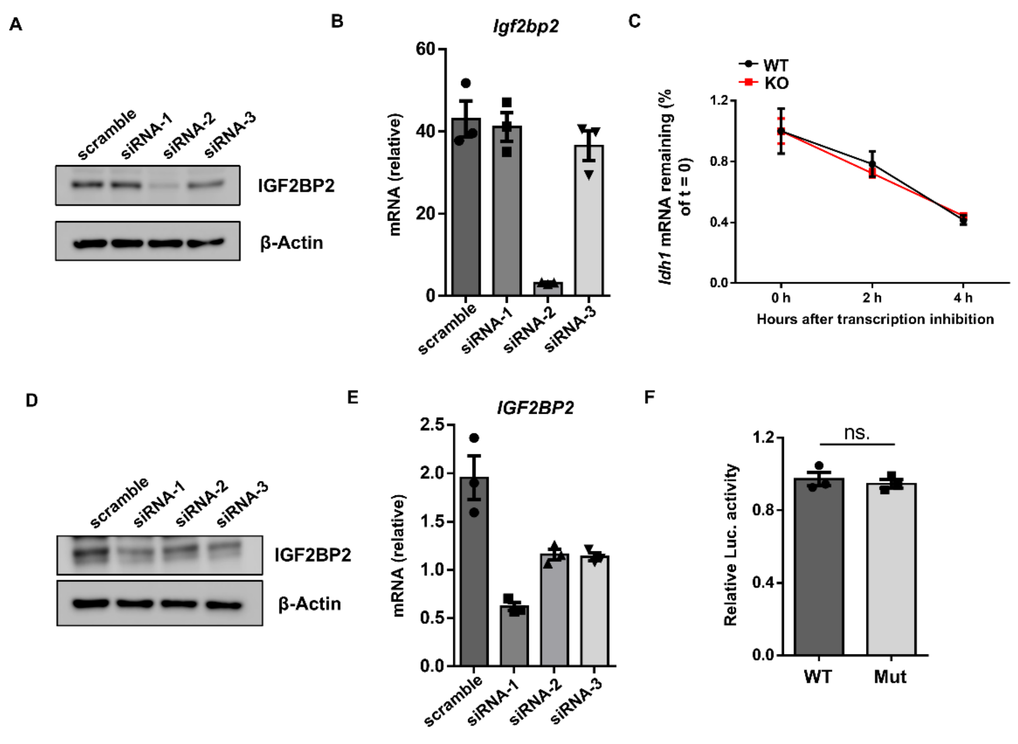

Fig. S5. IGF2BP2 modulates *Idh1* mRNA stability and translation. A-B Knockdown efficiency of IGF2BP2-specific siRNAs in BMDMs at the protein (A) and mRNA (B) levels **C** RT-qPCR analysis of *Idh1* mRNA stability in BMDMs treated with 5 μg/mL Actinomycin D for 0, 2, and 4 hours following IGF2BP2 knockdown. **D-E** Knockdown efficiency of IGF2BP2-specific siRNAs in HEK293T cells at the protein (D) and mRNA (E) levels **F** Relative luciferase activities of HEK293T cells co-transfected with WT or mutant plasmid and IGF2BP2 siRNA. Data were exhibited as mean ± SEM and analyzed by unpaired Student's *t* test. ns. no significance.

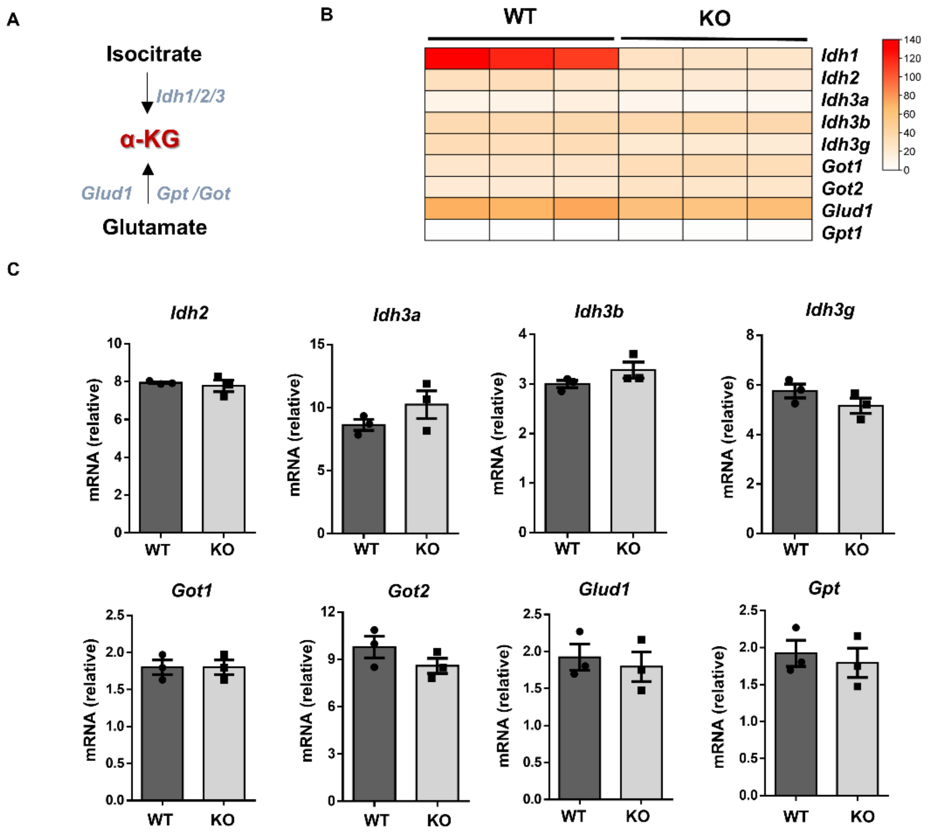

Fig. S6. Expression profile of the genes critical for α-KG production. A Alpha-KG productions are mainly from isocitrate and glutamate. **B** Heatmap of the vital enzyme genes related to α-KG production. **C** RT-qPCR analysis of abovementioned genes. Data were exhibited as mean ± SEM and analyzed by unpaired Student's *t* test. All results presented no significant differences.

Table S1. Clinical characteristics of subjects

	**Non-obese**	**Obese**
**Number**	12	46
**Male/ female (n)**	2/10	15/31
**Age (years)**	32.17 ± 2.007	33.65 ± 1.173
**BMI (kg/m^2)**	27.75 ± 0.9062	38.25 ± 0.9697
**Fasting Bood Glucose (mmol/L)**	4.576 ± 0.1298	6.290 ± 0.3673
**Insulin (uU/ml)**	16.29 ± 2.795	35.50 ± 4.017
**c-peptide (nmol/L)**	1.810 ± 0.3674	1.533 ± 0.1034
**HbA1c (%)**	5.790 ± 0.4661	5.950 ± 0.1423
**Triglyceride (mmol/l)**	1.184 ± 0.1512	1.588 ± 0.1078
**Total cholesterol (mmol/l)**	4.781 ± 0.2800	5.125 ± 0.1727
**LDL (mmol/L)**	2.686 ± 0.1375	3.256 ± 0.1488
**HDL (mmol/L)**	1.198 ± 0.08451	1.193 ± 0.04704

Data were exhibited as mean ± SEM and analyzed by unpaired Student's *t* test.

Table S2. Primer sequences for RT-qPCR

Gene	Forward (5'-3')	Reverse (5'-3')
Nos2	GTTCTCAGCCCAACAATACAAGA	GTGGACGGGTCGATGTCAC
Il1b	TACGGACCCCAAAAGATGA	TGCTGCTGCGAGATTTGAAG
Tnf	ACTGAACTTCGGGGTGATCG	GGCTACAGGCTTGTCACTCG
Arg1	CTCCAAGCCAAAGTCCTTAGAG	AGGAGCTGTCATTAGGGACATC
Ym1	CAGGTCTGGCAATTCTTCTGAA	GTCTTGCTCATGTGTGTAAGTGA
Retnla	CTGGGTTCTCCACCTCTTCA	TGCTGGGATGACTGCTACTG
β-Actin	AGAGGGAAATCGTGCGTGAC	CAATAGTGATGACCTGGCCGT
Ucp1	AGGCTTCCAGTACCATTAGGT	CTGAGTGAGGCAAAGCTGATTT
Idh1	ATGCAAGGAGATGAAATGACACG	GCATCACGATTCTCTATGCCTAA
Cox5a	GGAAGACCCTAATCTAGTCCCG	GTTGGGGCATCGCTGACTC
Cox7a	GCTCTGGTCCGGTCTTTTAGC	GTACTGGGAGGTCATTGTCGG
Cox8b	TGTGGGGATCTCAGCCATAGT	AGTGGGCTAAGACCCATCCTG
Wtap	TAGACCCAGCGATCAACTTGT	CCTGTTTGGCTATCAGGCGTA
Igf2bp1	CGGCAACCTCAACGAGAGT	CGGCAACCTCAACGAGAGT

Igf2bp2	GTCCTACTCAAGTCCGGCTAC	CATATTCAGCCAACAGCCCAT
Igf2bp3	CCTGGTGAAGACGGGCTAC	TCAACTTCCATCGGTTTCCCA
***WTAP* (human)**	CTTCCCAAGAAGGTTCGATTGA	TCAGACTCTCTTAGGCCAGTTAC
***IDH1* (human)**	TGTGGTAGAGATGCAAGGAGA	TTGGTGACTTGGTCGTTGGTG
***IGF2BP2* (human)**	AGTGGAATTGCATGGGAAAATCA	CAACGGCGGTTTCTGTGTC
Idh2	GGAGAAGCCGGTAGTGGAGAT	GGTCTGGTCACGGTTTGGAA
Idh3a	TGGGTGTCCAAGGTCTCTC	CTCCCACTGAATAGGTGCTTTG
Idh3b	TGGAGAGGTCTCGGAACATCT	AGCCTTGAACACTTCCTTGAC
Idh3g	GGTGCTGCAAAGGCAATGC	TATGCCGCCCACCATACTTAG
Got1	GCGCCTCCATCAGTCTTTG	ATTCATCTGTGCGGTACGCTC
Got2	GGACCTCCAGATCCCATCCT	GGTTTTCCGTTATCATCCCGGTA
Glud1	CCCAACTTCTTCAAGATGGTGG	GGTTTTCCGTTATCATCCCGGTA
Gpt	TCCAGGCTTCAAGGAATGGAC	CAAGGCACGTTGCACGATG
